# Man With Acute Kidney Injury and Lower Abdominal Pain

**DOI:** 10.1016/j.acepjo.2026.100421

**Published:** 2026-05-27

**Authors:** Sarah Uhranowsky, Dylan Perez, Yosef Berlyand

**Affiliations:** Department of Emergency Medicine, Alpert Medical School, Brown University, Providence, Rhode Island, USA

**Keywords:** acute kidney injury, hernia, postrenal obstruction, abdominal pain, genitourinary emergency

## 1 Patient Presentation

A 52-year-old man with a past medical history of diabetes mellitus and hypertension presented to the emergency department (ED) with fever, abdominal pain, right leg swelling, and altered mental status. On physical examination, the patient had an obese abdomen with right-sided inguinal tenderness and no overlying skin changes. Laboratory evaluation demonstrated a leukocytosis (22.6 × 10^9^/L) and a creatinine of 2.24 mg/dL, which was previously normal. The patient was unable to provide a urine sample in the ED. He then underwent contrast-enhanced computed tomography (CT) of the abdomen and pelvis.

## 2 Diagnosis: Postrenal Obstructive Kidney Injury Due to Ureteral and Bladder Herniation

**Figure 1:**
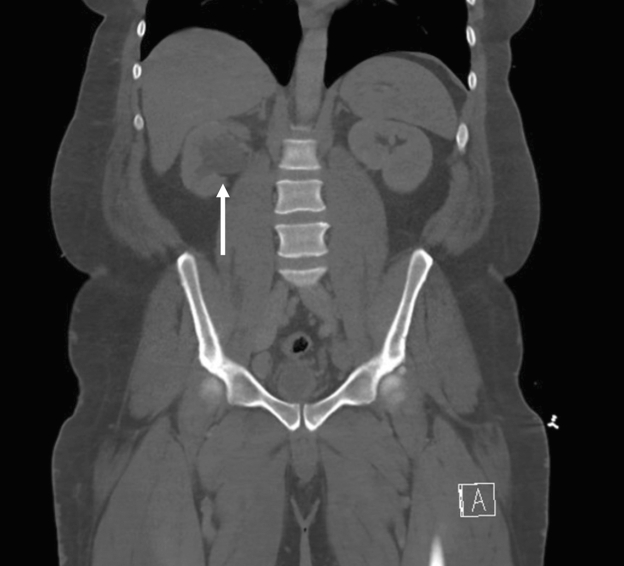
Computed tomography of the abdomen and pelvis demonstrating right hydronephrosis as denoted by the white arrow.

**Figure 2:**
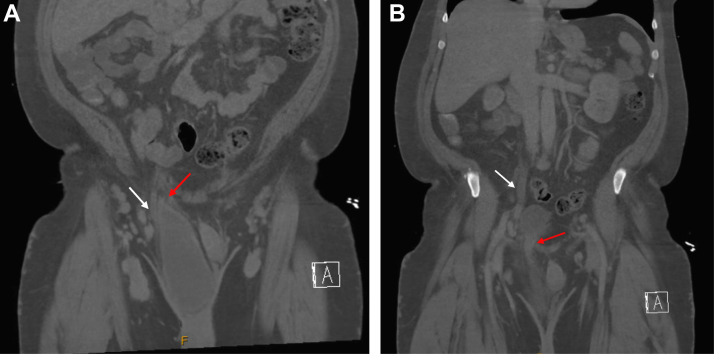
A and B, Computed tomography imaging of the abdomen and pelvis demonstrating ureteral and bladder herniation into an inguinal hernia sac. The white arrow denotes the ureter, and red arrow denotes the bladder as it passes into the hernia sac.

The CT scan revealed right-sided hydroureteronephrosis that extended into an inguinal hernia, which contained most of the bladder, including the right ureteral insertion site (Figs 1 and 2). The hernia was reduced at bedside, after which the patient was able to produce urine. The inpatient team planned a surgical repair while he was hospitalized, though this was ultimately delayed to the outpatient setting due to increased perioperative risks associated with an unrelated diagnosis of new pulmonary embolism while hospitalized.

Inguinal hernias are common with approximately 20 million repairs performed annually worldwide.^1^ Ureter and bladder involvement in the hernia is rare, with bladder herniation occurring in 1% to 4% of cases.^2^ Ureteral herniation has been reported ∼ 140 times in the literature through 2009,^1^ with acute kidney injury (AKI) secondary to herniation reported in a limited number of case reports.^3^ This case represents a rare cause of postrenal obstructive AKI due to ureteral and bladder herniation within an inguinal hernia sac.

## Funding and Support

By *JACEP Open* policy, all authors are required to disclose any and all commercial, financial, and other relationships in any way related to the subject of this article as per ICMJE conflict of interest guidelines (see www.icmje.org). The authors have stated that no such relationships exist.

## Conflicts of Interest

Dr Berlyand reports unpaid consulting activities for Esplanade Ventures and service on an emergency department steering committee for Cleerly Inc. These activities are unrelated to the clinical content of this Images in Emergency Medicine submission. All other authors have affirmed they have no conflicts of interest to declare.
